# Central role of c-Src in NOX5- mediated redox signalling in vascular smooth muscle cells in human hypertension

**DOI:** 10.1093/cvr/cvab171

**Published:** 2021-07-28

**Authors:** Livia L Camargo, Augusto C Montezano, Misbah Hussain, Yu Wang, Zhiguo Zou, Francisco J Rios, Karla B Neves, Rheure Alves-Lopes, Fazli R Awan, Tomasz J Guzik, Thomas Jensen, Richard C Hartley, Rhian M Touyz

**Affiliations:** Institute of Cardiovascular and Medical Sciences, University of Glasgow, 126 University Place, Glasgow G12 8TA, UK; Institute of Cardiovascular and Medical Sciences, University of Glasgow, 126 University Place, Glasgow G12 8TA, UK; Diabetes and Cardio-Metabolic Disorders Laboratory, Health Biotechnology Division, National Institute for Biotechnology and Genetic Engineering (NIBGE), Jhang Road, P.O. Box. 577, Faisalabad, Pakistan; Institute of Cardiovascular and Medical Sciences, University of Glasgow, 126 University Place, Glasgow G12 8TA, UK; Institute of Cardiovascular and Medical Sciences, University of Glasgow, 126 University Place, Glasgow G12 8TA, UK; Institute of Cardiovascular and Medical Sciences, University of Glasgow, 126 University Place, Glasgow G12 8TA, UK; Institute of Cardiovascular and Medical Sciences, University of Glasgow, 126 University Place, Glasgow G12 8TA, UK; Institute of Cardiovascular and Medical Sciences, University of Glasgow, 126 University Place, Glasgow G12 8TA, UK; Diabetes and Cardio-Metabolic Disorders Laboratory, Health Biotechnology Division, National Institute for Biotechnology and Genetic Engineering (NIBGE), Jhang Road, P.O. Box. 577, Faisalabad, Pakistan; Institute of Cardiovascular and Medical Sciences, University of Glasgow, 126 University Place, Glasgow G12 8TA, UK; WestCHEM School of Chemistry, University of Glasgow, University Avenue, G12 8QQ Glasgow, UK; WestCHEM School of Chemistry, University of Glasgow, University Avenue, G12 8QQ Glasgow, UK; Institute of Cardiovascular and Medical Sciences, University of Glasgow, 126 University Place, Glasgow G12 8TA, UK

**Keywords:** NOX5, Hypertension, Oxidative stress, Vascular smooth muscle cells

## Abstract

**Aims:**

NOX-derived reactive oxygen species (ROS) are mediators of signalling pathways implicated in vascular smooth muscle cell (VSMC) dysfunction in hypertension. Among the numerous redox-sensitive kinases important in VSMC regulation is c-Src. However, mechanisms linking NOX/ROS to c-Src are unclear, especially in the context of oxidative stress in hypertension. Here, we investigated the role of NOX-induced oxidative stress in VSMCs in human hypertension focusing on NOX5, and explored c-Src, as a putative intermediate connecting NOX5-ROS to downstream effector targets underlying VSMC dysfunction.

**Methods and results:**

VSMC from arteries from normotensive (NT) and hypertensive (HT) subjects were studied. NOX1,2,4,5 expression, ROS generation, oxidation/phosphorylation of signalling molecules, and actin polymerization and migration were assessed in the absence and presence of NOX5 (melittin) and Src (PP2) inhibitors. NOX5 and p22phox-dependent NOXs (NOX1–4) were down-regulated using NOX5 siRNA and p22phox-siRNA approaches. As proof of concept in intact vessels, vascular function was assessed by myography in transgenic mice expressing human NOX5 in a VSMC-specific manner. In HT VSMCs, NOX5 was up-regulated, with associated oxidative stress, hyperoxidation (c-Src, peroxiredoxin, DJ-1), and hyperphosphorylation (c-Src, PKC, ERK1/2, MLC_20_) of signalling molecules. NOX5 siRNA reduced ROS generation in NT and HT subjects. NOX5 siRNA, but not p22phox-siRNA, blunted c-Src phosphorylation in HT VSMCs. NOX5 siRNA reduced phosphorylation of MLC_20_ and FAK in NT and HT. In p22phox- silenced HT VSMCs, Ang II-induced phosphorylation of MLC_20_ was increased, effects blocked by melittin and PP2. NOX5 and c-Src inhibition attenuated actin polymerization and migration in HT VSMCs. In NOX5 transgenic mice, vascular hypercontractilty was decreased by melittin and PP2.

**Conclusion:**

We define NOX5/ROS/c-Src as a novel feedforward signalling network in human VSMCs. Amplification of this system in hypertension contributes to VSMC dysfunction. Dampening the NOX5/ROS/c-Src pathway may ameliorate hypertension-associated vascular injury.

## Graphical Abstract

**Figure:**
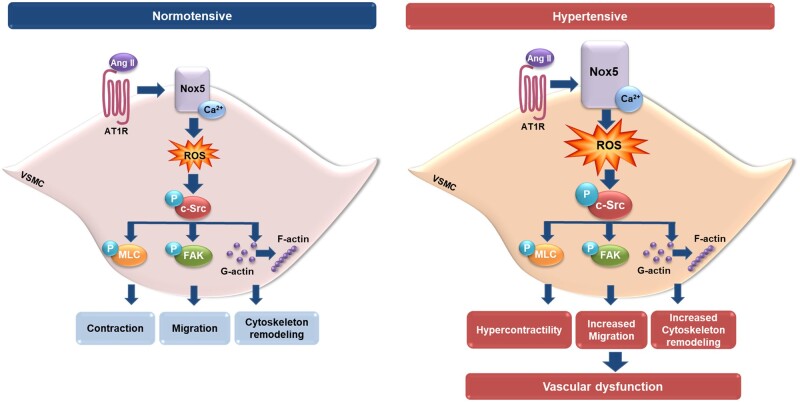

## 1. Introduction

Hypertension is a complex disorder and important risk factor for cardiovascular diseases, such as stroke, myocardial infarction, and heart failure, major causes of premature death.^1^ Moreover, patients with underlying hypertension appear to have an increased risk for adverse outcomes with coronavirus disease 2019.^2^ Accordingly, the societal health burden of hypertension and its complications is enormous. Therefore, there is an urgent need to better understand molecular mechanisms underlying hypertension so that mechanism-targeted and disease-specific therapies can be developed.

Small resistance arteries are critically involved in blood pressure control as they play a central role in peripheral resistance. In hypertension, resistance arteries undergo increased contractility, endothelial dysfunction, inflammation, and structural remodelling that are involved in both cause and consequence of increased blood pressure.^3^ The search for molecular mechanisms underlying vascular alterations associated with hypertension identified NADPH oxidase (NOX)-derived reactive oxygen species (ROS) as key players.

The NOX family of enzymes comprises seven isoforms: NOX1–5, DUOX1, and DUOX2. Activation of NOX1–4 involves interaction with the cell membrane-associated subunit p22phox, whereas NOX5 is p22phox-independent. All NOX isoforms are transmembrane proteins that transfer an electron from NADPH to O_2_, producing superoxide anion ($O_{2}^{•}$^−^), that is rapidly converted to hydrogen peroxide (H_2_O_2_).^4^ These ROS are now considered important second messengers in vascular signalling. Through oxidative post-translational modifications, ROS influence activation of signalling pathways regulating diverse cellular functions, such as differentiation, survival, growth, contraction, migration, and production of extracellular matrix.^5^

However, in pathological conditions, including hypertension, high levels of ROS lead to oxidative stress and aberrant redox signalling, resulting in abnormal vascular function. Alterations in ROS production and oxidative stress in hypertension are dependent on activation of vascular NOX1, NOX2, and NOX4, as demonstrated in almost all experimental models.^6^ The role of NOX5 in hypertension-associated vasculopathy is unclear because rodents, the most common model used to study hypertension, lack *NOX5* gene. NOX5 is a unique NOX isoform. Unlike NOX1–4, it does not require binding to membrane (p22phox) or cytosolic subunits (p47phox, p67phox, NoxO1, NoxA1, Rac) for its activation. NOX5 is activated by calcium (Ca^2+^) binding to its N-terminal EF hands and is regulated by post-translational modifications.^7^ NOX5 is expressed in endothelial cells and vascular smooth muscle cells (VSMCs) in the human vasculature and responds to agonists that contribute to cardiovascular diseases, such as angiotensin II (Ang II) and endothelin-1 (ET-1).^8^^,^^9^ We demonstrated that NOX5 is important in redox-sensitive contraction in VSMCs.^10^

NOX5 has been implicated in cardiovascular diseases, such as coronary artery disease, atherosclerosis, and stroke as well as diabetic retinopathy, nephropathy, and vasculopathy.^7^ Increasing evidence supports a role for NOX5 in hypertension. Blood pressure is increased in NOX5 knockin mice.^11^ Additionally, NOX5 expression and activity were significantly higher in renal proximal tubule cells from hypertensive (HT) subjects.^12^ Of significance, genome-wide association studies identified NOX5 as an important gene associated with blood pressure.^13^ There is still a paucity of information on the molecular mechanisms that regulate vascular NOX5 and the downstream signalling pathways and networks through which NOX5-ROS mediate effects, particularly in human hypertension, is elusive.

Of the numerous kinases that function as a signalling hub in VSMCs is c-Src, an intracellular non-receptor tyrosine kinase. Unlike other Src-family kinases, c-Src is highly expressed in VSMCs and is activated by Ang II, aldosterone, ET-1, and growth factors. c-Src polymorphisms are associated with hypertension^14^ and we showed that c-Src-deficient mice are resistant to Ang II-induced hypertension.^15^ c-Src regulates multiple downstream targets including MAPK, PI3K, and protein kinase C (PKC) and plays an important role in VSMC contraction, growth, cytoskeletal organization, and migration.^16^ In addition to regulation by tyrosine (de)phosphorylation, c-Src activity is influenced by intracellular ROS through direct cysteine sulfenylation.^17^ Oxidative stress is associated with increased c-Src-mediated signalling and VSMC dysfunction, processes that contribute to vascular damage in hypertension. While c-Src is downstream of NOX/ROS, it may also be upstream of NOX, since NOX1 and NOX2 are regulated through c-Src-dependent processes.^18^

The NOX/ROS/c-Src pathway may be a key process linking redox and kinase signalling in VSMCs. However, the exact interplay between these elements and the role of NOX5 in c-Src signalling is unknown, especially in the context of human hypertension. Therefore, we investigated whether NOX5-derived ROS influences c-Src and its downstream signalling in human hypertension, using VSMCs isolated from small resistance arteries from normotensive (NT) and HT subjects.

## 2. Methods

### 2.1 Human vascular tissue

Ethics approval was obtained from the West of Scotland Research Ethics Service (WS/12/0294) and the research ethics board of the Ottawa Hospital Research Institute (OHRI), Canada (#997392132). Written informed consent was obtained for all study participants in accordance with the Declaration of Helsinki.

Vascular tissue was obtained from NT (*n* = 13; 128/78 ± 3/2 mmHg) and HT subjects (*n* = 13; 144/82 ± 6/2 mmHg) undergoing elective maxillofacial surgery at the Craniofacial/Oral and Maxillofacial Unit, Queen Elizabeth University Hospital, Glasgow (Supplementary *Table S1*). Following surgery, isolated small arteries dissected from excess surgical tissue from the neck or face were cleaned from adipose tissue and processed for western blot. Gluteal biopsies of subcutaneous fat were obtained under local anaesthetic from NT (*n* = 10; 120/74 ± 3/3 mmHg) and HT (*n* = 5, 147/93 ± 6/3 mmHg) volunteers at the OHRI (Supplementary *Table S2*). Small arteries were dissected from these biopsies and used for primary culture of VSMCs.

### 2.2 Primary culture of VSMCs from human small arteries

Primary VSMCs were isolated from small arteries obtained from gluteal biopsies, by enzymatic digestion, as we previously described.^19^ Cell identity was confirmed by western blot of VSMC and fibroblasts markers (Supplementary *Figure S1*).

### 2.3 VSMC‐specific NOX5 transgenic mice

All experimental protocols on mice were performed in accordance with the Ethical Principles in Animal Experimentation adopted by the West of Scotland Research Ethics Service and in accordance with the United Kingdom Animals Scientific Procedures Act 1986 and ARRIVE Guidelines and approved by the institutional ethics review committee (70/9021).

VSMC-specific NOX5-expressing mice were used to assess whether c-Src inhibition influences vascular function. The transgenic NOX5 mice have been previously characterized and described.^10^ Animals were euthanized by overdose of anaesthetic gas (isoflurane) followed by cervical dislocation. Small mesenteric arteries were isolated and used to assess vascular function by wire myography. Mesenteric arteries were used to investigate NOX5 dependent signalling.

### 2.4 NOX5 and p22phox down-regulation with siRNA

VSMCs were transfected with NOX5 (50 nmol/L) or p22phox siRNA (20 nmol/L) for 6 h. A sequence not homologous to any gene in the vertebrate transcriptome was used as control siRNA. After transfection, medium was replaced by growth medium and experiments were conducted 12–48 h after transfection.

### 2.5 Measurement of NADPH-dependent O_2_^−^ generation

Lucigenin-enhanced chemiluminescence assay was used to detect NADPH-dependent ROS generation in VSMC as we previously described.^20^

### 2.6 Measurement of vascular H_2_O_2_

H_2_O_2_ levels in VSMC were assessed using the Amplex Red H_2_O_2_/Peroxidase Assay Kit (Life Technologies) according to manufactureŕs instructions.

### 2.7 Measurement of O_2_•^−^ by electron paramagnetic resonance

Superoxide anion (O_2_•^−^) was measured by electron paramagnetic resonance as previously described.^21^

### 2.8 Immunoblotting

Total protein from vascular tissue and cells was resolved by SDS-PAGE, transferred onto a nitrocellulose membrane and incubated with protein-specific primary antibodies. Fluorescence-coupled antibodies were visualized by an infrared laser scanner. Images were quantified using the software Image Studio™ Lite.

### 2.9 Assessment of protein sulfenylation

Cells were scraped in lysis buffer supplemented with BCN-E-BCN, that specifically binds sulfonylated proteins.^22^ BCN-E-BCN was conjugated with biotin using a copper-free click reaction. Equal amounts of protein were incubated with streptavidin beads proteins were eluted in sample buffer and analysed by immunoblotting.

### 2.10 Determination of irreversible DJ-1 and Prx oxidation

Cell homogenates was analysed by immunoblotting using specific antibodies, which recognize the hyperoxidized cysteine (-SO_3_H) on DJ-1 and Prx (Prx-SO3).

### 2.11 Calcium (Ca^2+^) influx

Intracellular Ca^2+^ levels were measured in VSMCs from NT and HT subjects using the fluorescent Ca^2+^ indicator, Cal-520 acetoxymethyl ester (Cal-520/AM) using an inverted epifluorescence microscope with excitatory wavelengths of 490 and 488 nm. In some experiments, VSMCs were pre-treated with melittin, PP2, superoxide dismutase polyethylene glycol and catalase polyethylene glycol.

### 2.12 Cell migration

VSMCs migration was assessed by modified Boyden chamber assay. VSMCs were harvested and added to the insert (5 × 10^4^ cells/well), and culture medium with or without Ang II (100 nmol/L) was added to the chamber. After 16 h, non-migrating cells were removed from upper filter surfaces and the filter was washed, fixed, stained, and imaged. The number of cells that migrated was determined using Image J software (National Institutes of Health, USA).

### 2.13 Phalloidin staining

Cytoskeletal organization was assessed using phalloidin staining according to the manufacturer’s instructions. Briefly, VSMCs were grown in four-chamber slides, incubated with Ang II in the presence and absence of melittin and PP2, fixed with 4% paraformaldehyde and processed for phalloidin staining. Fluorescence imaging was performed using a 40× plan dry lens in a LSM500 confocal imaging system (Zeiss).

### 2.14 G-actin/F-actin assay

Filamentous actin (F-actin) and free globular-actin (G-actin) content were assessed from lysates of cells from NT and HT subjects using G-actin/F-actin *in vivo* assay according to the manufacturer’s recommendations. G-actin and F-actin samples were analysed by immunoblotting. G-actin to F-actin ratio was quantified by densitometry using the software Image Studio™ Lite.

### 2.15 Vascular function assessed by wire myography

Mesenteric resistance arteries from wild type (WT) and NOX5 transgenic mice (NOX5^+^SM22^+^) were dissected as previously described.^10^ U46619 (thromboxane A2 analogue) concentration–response curves were generated to evaluate vasoconstriction in arteries form WT and NOX5 mice. In some experiments, arteries were preincubated with melittin or PP2.

### 2.16 Statistical analysis

All results are reported as mean ± SEM. For comparisons between two groups *t*-test was used. For multiple comparisons one-way or two-way analysis of variance (ANOVA) followed by Bonferroni’s post-test was conducted where appropriated. Graphs were plotted in GraphPad Prism 8 software. *P* < 0.05 was considered significant.

## 3. Results

### 3.1 Oxidative stress and hyperoxidation of peroxiredoxin and DJ-1 in VSMCs from HT subjects

**Figure 1:**
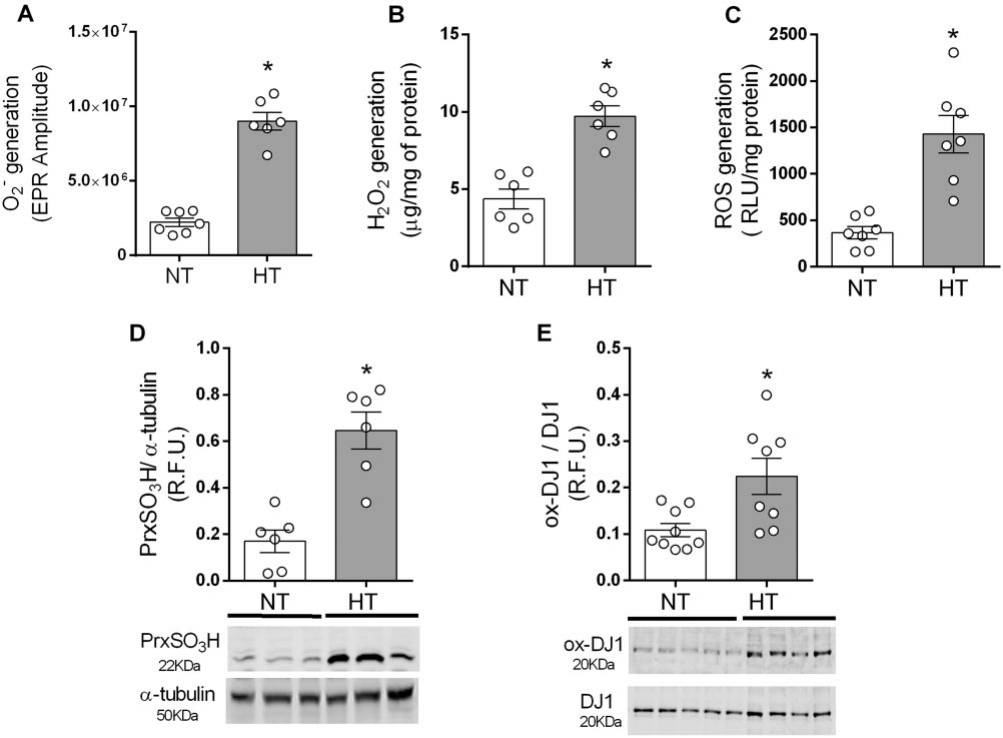
ROS generation and oxidation of signalling proteins are increased in HT subjects. Superoxide anion, H_2_O_2_, and NADPH-dependent ROS were measured by EPR (*A*), amplex red (*B*), and lucigenin-derived chemiluminescence (*C*). Irreversible protein oxidation was assessed by western blot of peroxiredoxin (PrxSO_3_H) hyperoxidation (*D*) and DJ-1 oxidation (*E*). α-Tubulin or total DJ-1 was used as loading control. Results are expressed as mean ±SEM of *n*=6–8. Statistical significance was determined by unpaired Student’s *t*-test. *P<0.05 vs. NT.

Levels of O_2_^−^ and H_2_O_2_ were increased in HT compared to NT VSMCs (*Figure 1A and B*). This was associated with increased NADPH-dependent ROS production in HT (*Figure 1C*). To confirm specificity of ROS measurements, ROS generation was measured in the absence and presence of antioxidant enzymes superoxide dismutase (SOD) and catalase (Supplementary *Figure* *S2*). Treatment of cells with pegylated SOD reduced superoxide generation (Supplementary *Figure* *S2A* *and* *B*) and NADPH-dependent ROS production induced by Ang II in cells from both NT and HT (Supplementary *Figure* *S2C* *and* *D*), but had no effect on H_2_O_2_ generation (Supplementary *Figure* *S2E* *and* *F*). Treatment with pegylated catalase resulted in a decrease in both H_2_O_2_ and NADPH-dependent ROS production (Supplementary *Figure* *S2C–F*).

Once generated, ROS induce post-translational oxidative modification of proteins. Cysteine residues in proteins are highly redox-sensitive and reversible cysteine oxidation provides a mechanism of redox switching in protein regulation. High ROS levels result in irreversible oxidation, such as formation of sulfinic and sulphonic acid on cysteine residues (SO_2_H, SO_3_H), that translates into inactivation or change of protein function. To investigate irreversible protein oxidation levels in VSMCs, oxidized peroxiredoxins (Prx-SO_3_), and DJ-1 (oxDJ-1) were assessed by western blot. As shown in *Figure 1D and E*, irreversible oxidation of both proteins was higher in HT vs. NT VSMCs.

### 3.2 Increased NOX5 expression in HT vessels and VSMCs

**Figure 2:**
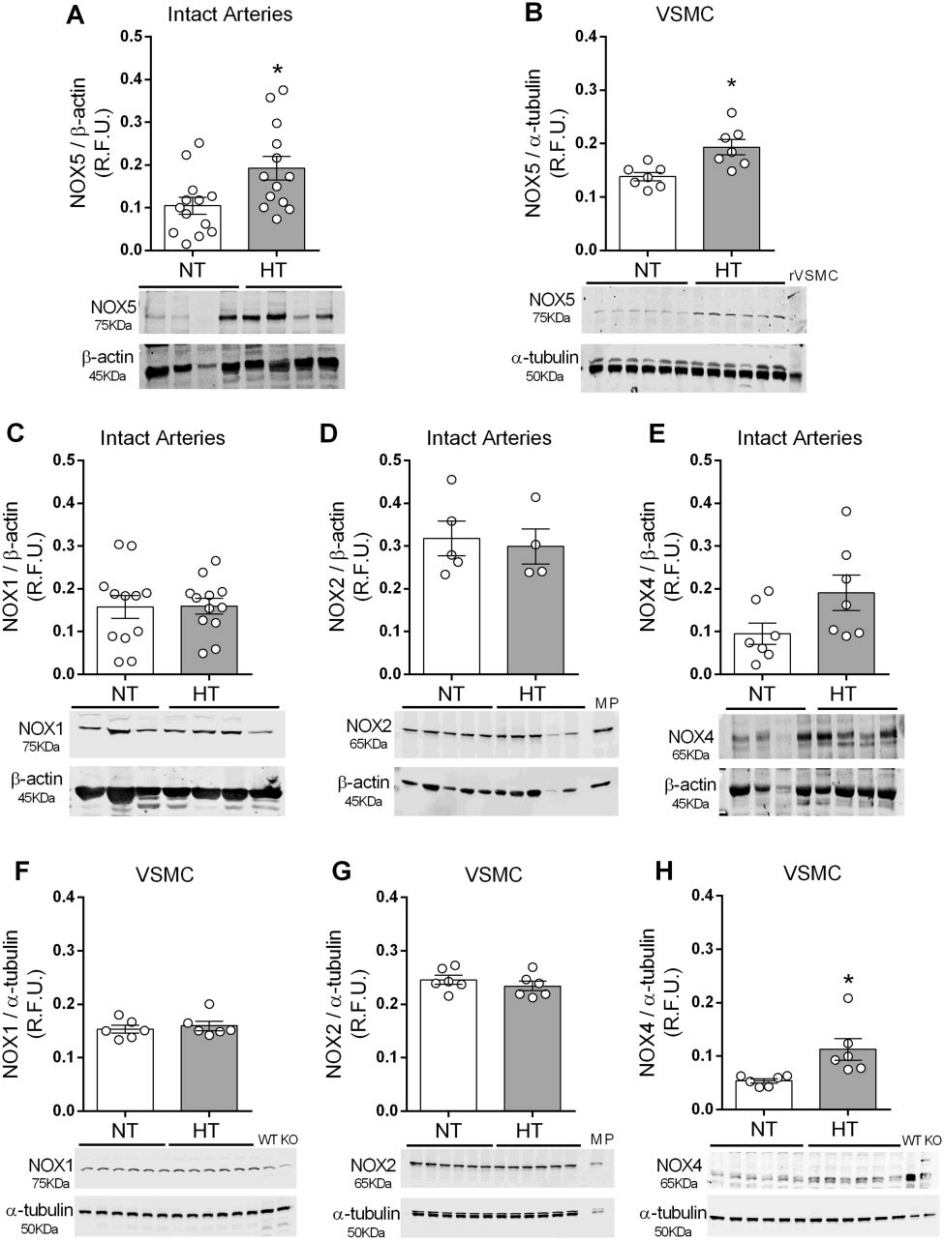
NOX isoform expression in intact vessels and VSMCs from NT and HT subjects. Expression of NOX5 was detected by western blot in intact arteries (*A*) and VSMC (*B*) from NT and HT subjects. Expression of NOX1, NOX2, and NOX4 was detected in small arteries (*C*, *D,* and *E*, respectively) and in VSMCs (*F*, *G,* and *H*, respectively) from NT and HT subjects. α-Tubulin or β-actin was used as loading control. Nox1 and Nox4 knockout (KO) and wild-type (WT) mice were used as positive and negative control. Macrophages (MP) isolated from mice bone marrow were used as positive control for Nox 2. Results are expressed as mean ±SEM of *n*=4–13. Statistical significance was determined by unpaired Student’s *t*-test. *P<0.05 vs. NT.

To characterize vascular NOX isoforms, we assessed expression of NOX1, 2, 4, and 5 in VSMCs and small arteries from NT and HT subjects. Of all the NOX isoforms, expression of NOX5 was significantly increased in intact arteries (*Figure 2A*) and VSMCs (*Figure 2B*) from HT vs. NT subjects.

NOX1, NOX2, and NOX4 were detected in vessels and VSMC from both groups (*Figure 2C–H*). NOX1, NOX2, and NOX4 were present in intact arteries, with no significant difference in abundance between NT and HT groups (*Figure 2C–E*). Expression of NOX4, but not NOX1 or NOX2, was increased in HT VSMCs compared to NT cells (*Figure 2F–H*). Expression of NOX1–4 subunit, p22phox, was not altered in cells from HT compared to NT subjects (Supplementary *Figure S3*).

### 3.3 NOX5 an important ROS-generating NOX isoform in human VSMCs

**Figure 3:**
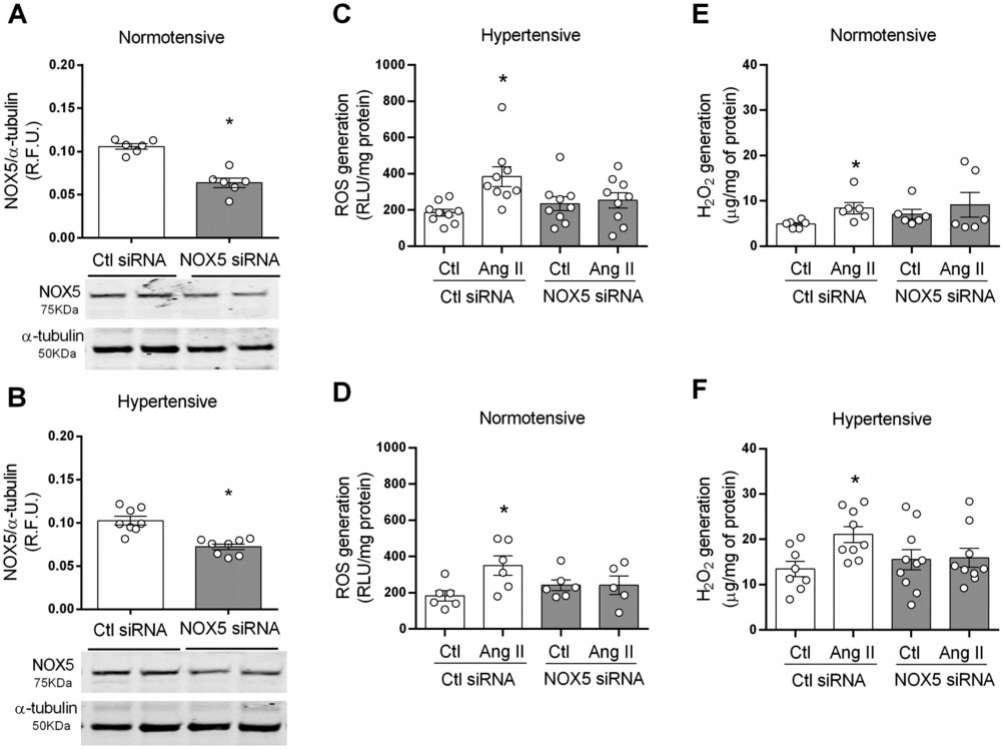
NOX5 is involved in Ang II-induced ROS generation in human VSMC. (*A*) Cells were stimulated with Ang II (100 nmol/L) for 5 min in VSMCs transfected with NOX5 or control (Ctl) siRNA. NOX5 expression in VSMC from NT (*A*) and HT (*B*) subjects detected by western blot. NADPH-dependent ROS and H_2_O_2_ were measured by lucigenin-derived chemiluminescence and amplex red in VSMCs from NT (*C* and *E*, respectively) and HT (*D* and *F*, respectively) subjects. Results are expressed as mean±SEM of 6–9 separate experiments. Statistical significance was determined by unpaired Student’s *t*-test for comparisons between two groups and one-way ANOVA followed by Bonferroni’s post-test for multiple comparisons. *P<0.05 vs. Ctl.

Having demonstrated that NOX5 was up-regulated in HT subjects, we next questioned its role on oxidative stress in HT. To address this NOX5 was silenced using NOX5 siRNA in cells from both NT and HT subjects (*Figure 3A and B*). NADPH-dependent ROS and H_2_O_2_ levels in basal and Ang II-stimulated cells were measured. In cells transfected with control siRNA Ang II increased both NADPH-dependent ROS and H_2_O_2_ levels. However, Ang II failed to increase ROS generation in cells transfected with NOX5 siRNA (*Figure 3C–F*). Hence, NOX5 is likely an important player in basal ROS production in NT and HT VSMCs.

### 3.4 Increased phosphorylation of c-Src and up-regulation of signalling pathways in VSMC from HT subjects

c-Src is a master upstream regulator in VSMCs and plays a key role in Ang II-induced contraction and growth signalling pathways. Phosphorylation at Tyr416 mediates c-Src activation, while Tyr512 phosphorylation maintain c-Src in the inactive state. In cells from HT subjects, c-Src phosphorylation at Tyr416 was increased while at Tyr512 was decreased compared to NT (Supplementary *Figure* *S4A* *and* *B*). Other molecular players involved in VSMC function include PKC and extracellular signal-regulated kinase 1 and 2 (ERK1/2), important in cell growth, and myosin light chain (MLC), critically involved in myosin–actin interaction and contraction initiation. In cells from HT subjects phosphorylation of PKC, ERK 1/2, and MLC was increased compared to NT (Supplementary *Figure* *S4C–E*). Another key mechanism involved in vascular contraction is the increase in Ca^2+^ influx in response to stimuli. Here, we assessed Ca^2+^ influx induced by Ang II in cells from NT and HT subjects. As shown in Supplementary *Figure* *4F and* *G*, Ang II increased intracellular Ca^2+^ levels in both groups, with a greater magnitude of increase in cells from the HT vs. NT group.

To investigate the role of ROS in vascular signalling, we assessed Ang II-induced signalling in the absence and presence of antioxidant enzymes SOD and catalase (Supplementary *Figure* *S5A–F*). In basal conditions, there was no effect of antioxidant enzymes treatment on c-Src, PKC, and MLC phosphorylation in NT VSMCs. However, treatment of HT cells with pegylated SOD reduced c-Src and MLC phosphorylation, suggesting a role for superoxide in c-Src and MLC increased activation in HT subjects. In cells stimulated with Ang II, pegylated catalase but not SOD reduced c-Src activation in NT subjects whereas both antioxidant enzymes reduced c-Src phosphorylation in HT. Ang II-induced activation of PKC was reduced by both SOD and catalase, while MLC phosphorylation was reduced only by pegylated SOD in cells from NT and HT subjects.

In addition, we assessed Ang II-induced Ca^2+^ influx in the absence and presence of antioxidant enzymes SOD and catalase (Supplementary *Figure* *S6*). Treatment of cells with pegylated SOD or pegylated catalase resulted in a decrease in Ang II-stimulated Ca^2+^ transients in HT VSMCs but not in NT VSMCs. Altogether, these results suggests that ROS contributes to Ang II signalling in human VSMC and that oxidative stress contributes to increased VSMC signalling in HT.

### 3.5 Nox5/ROS is involved in Ang II-induced c-Src oxidation and phosphorylation in VSMCs from HT subjects

**Figure 4:**
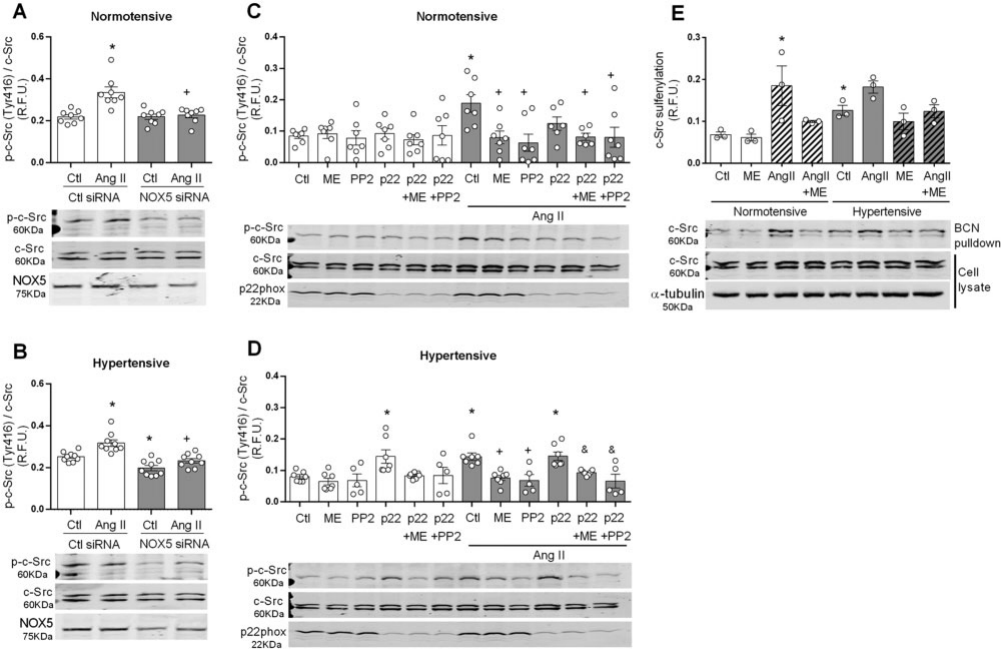
NOX5 regulates activation of c-Src in human VSMC. Cells were stimulated with Ang II (100 nmol/L) for 5 min in VSMCs transfected with NOX5, p22 phox or control (Ctl) siRNA. Some experiments were performed in the presence and absence of melittin (NOX5 inhibitor, 10 nmol/L) and PP2 (Src inhibitor, 10 μmol/L). Phosphorylation of c-Src on Tyr416 was detected by western blot in NT (*A*) and HT (*B*) VSMCs transfected with NOX5 siRNA. To exclude Nox1-4 effects, phosphorylation of c-Src was detected by western blot in cells from NT (*C*) and HT (*D*) VSMCs transfected with p22phox siRNA. Sulfenylated c-Src (*E*) was pulled down by affinity capture using the BCN-E-BCN probe. Protein quantification was normalized by total c-Src or α-tubulin. Results are expressed as mean±SEM of 3–9 separate experiments. Statistical significance was determined by unpaired Student’s *t*-test for comparisons between two groups and one-way ANOVA followed by Bonferroni’s post-test or two-way ANOVA for multiple comparisons. *P<0.05 vs. control (Ctl), ^+^*P*<0.05 vs. Ctl + Ang II and ^&^*P*<0.05 vs. p22 + Ang II.

Having observed up-regulation of c-Src and NOX5 in HT VSMCs and that ROS is involved in c-Src activation, we questioned their inter-relationship. Therefore, we assessed the role of NOX5 in c-Src phosphorylation on Tyr-416 (*Figure 4*). NOX5 silencing resulted in reduced basal activation of c-Src only in cells from HT subjects (*Figure 4A and B*). Ang II-induced c-Src activation was reduced in cells transfected with NOX5 siRNA compared to control in both NT and HT subjects (*Figure 4A and B*).

Furthermore, since NOX1–4 activation is dependent on p22phox, we used silencing of this subunit to inhibit NOX1–4 activity (*Figure 4C and D*). In some experiments, melittin that inhibits NOX5 by interaction with its N-terminal^23^ was used as NOX5 inhibitor. In NT cells, Ang II-induced an increase in phosphorylation of c-Src, an effect blocked by melittin and by the Src inhibitor PP2 (*Figure 4C*). In NT cells treated with p22phox siRNA, Ang II partially increased c-Src activation, effects that were reduced when melittin was added. In HT VSMCs, p22phox silencing resulted in increased c-Src activation in basal and Ang II-stimulated conditions, effects reduced by melittin (*Figure 4D*). These results indicate that increased phosphorylation of c-Src in HT VSMCs involves primarily NOX5.

Activation of c-Src is regulated by oxidation of critical cysteines as sulfenylation of c-Src results in exposure of Tyr-416 promoting its phosphorylation.^17^ Next, using the biotinylated probe BCN-E-BCN to trap sulfenylated proteins and performing affinity capture assay using streptavidin beads, we pulled down and identified sulfenylated c-Src in cells from NT and HT (*Figure 4E*). c-Src oxidation was higher in VSMCs from HT subjects compared to NT. Ang II stimulation promoted sulfenylation of c-Src in both NT and HT VSMCs, an effect attenuated by melittin.

### 3.6 c-Src is involved in ROS generation in hVSMC but not in NOX5 phosphorylation

In addition to being regulated by ROS, c-Src may play a role in Ang II-induced ROS generation. To determine whether NADPH-mediated ROS generation involves c-Src, Ang II effects were assessed in VSMCs pre-treated with the Src inhibitor, PP2. As shown in Supplementary *Figure* *S7A–D*, PP2 inhibited Ang II-induced O_2_^−^ and H_2_O_2_ production in NT and HT subjects, suggesting a role for c-Src in NOX-derived ROS in human VSMCs.

### 3.7 c-Src is a key player in Ang II-NOX5 mediated vascular signalling in hypertension

**Figure 5:**
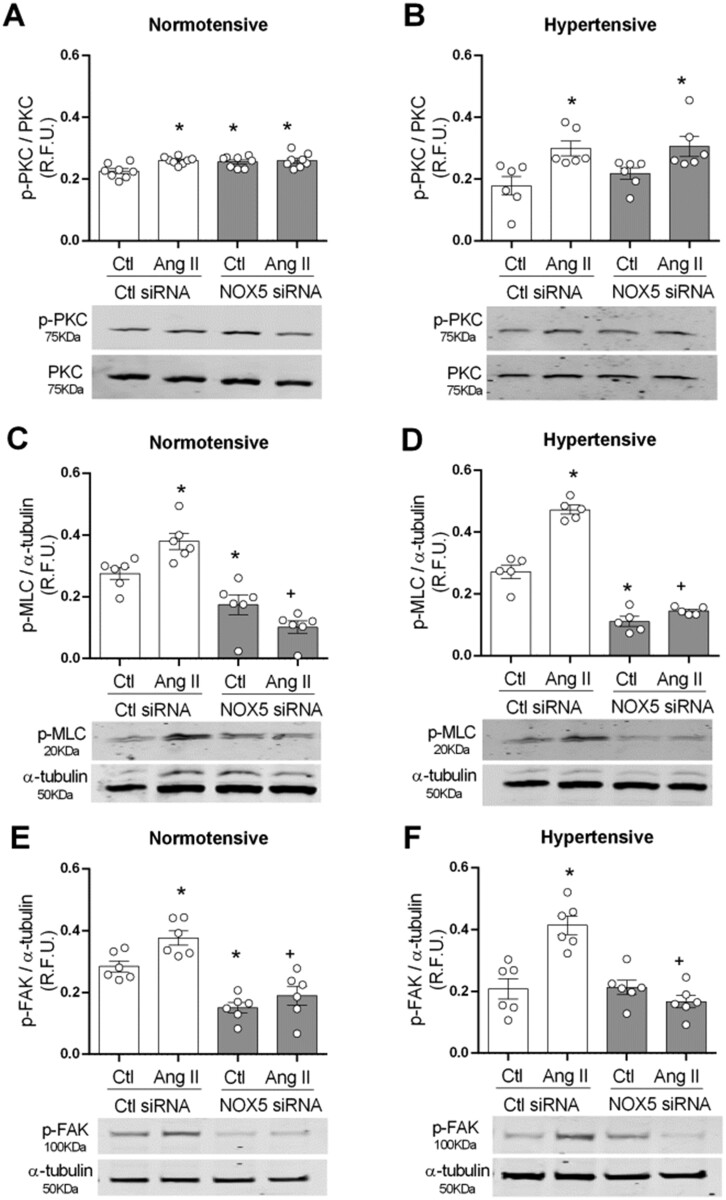
NOX5 induced activation of contractile and cytoskeletal signalling pathways in HT subjects is c-Src dependent. (*A*) NOX5 silenced cells were stimulated with Ang II (100 nmol/L) for 5 min. PKC (*A* and *B*, respectively), MLC (*C* and *D*, respectively), and FAK (*E* and *F*, respectively) were detected by western blot in cells from NT and HT subjects. Western Total PKC or α-tubulin was used as loading control. Results are expressed as mean±SEM of 5–8 separate experiments. Statistical significance was determined by one-way ANOVA followed by Bonferroni’s post-test for multiple comparisons. *P<0.05 vs. control (Ctl) and ^+^*P*<0.05 vs. Ctl + Ang II.

Next, we investigated the role of NOX5/c-Src interplay in the activation of contractile and structural signalling pathways by assessing phosphorylation of PKC and MLC and focal adhesion kinase (FAK) in basal and Ang II-stimulated VSMCs transfected with NOX5 siRNA (*Figure 5*).

In NT and HT VSMCs, Ang II induced an increase in PKC activation (*Figure 5A and B*). NOX5 silencing resulted in increase in PKC activation in basal conditions in cells from NT subjects. In HT, NOX5 silencing had no effect on PKC phosphorylation in basal conditions or after Ang II stimulation, suggesting NOX5 is not involved in PKC activation in human VSMC. MLC activation was also observed after Ang II treatment in cells from both groups (*Figure 5C and D*). NOX5 silencing resulted in reduced phosphorylation of MLC in basal conditions and in Ang II-stimulated cells from both NT and HT subjects, suggesting an important role for NOX5 in MLC activation in hVSMC. In addition, Ang II increased activation of FAK in hVSMC (*Figure 5E and F*). NOX5 silencing decreased basal FAK phosphorylation in VSMC from NT subjects. Ang II failed to induce FAK activation after Ang II stimulation in both NT and HT subjects.

To confirm the role of NOX5 in MLC activation NOX1–4 was inhibited by p22phox siRNA. Additionally, cells transfected with p22 siRNA were treated with melittin or PP2 to confirm the role of NOX5 and NOX5/Src interplay in VSMC signalling (Supplementary *Figure S8A* *and* *B*). In NT, p22phox silencing increase MLC phosphorylation in basal conditions, an effect not observed with NOX5 or c-Src inhibition. Ang II-induced MLC phosphorylation in control and p22phox siRNA but failed to induce MLC activation in cells treated with melittin or PP2. Similarly, in HT NOX5 or c-Src inhibition reduced basal and Ang II-induced MLC activation in control and p22phox-silenced cells. Altogether these results suggest that NOX5/c-Src interplay is involved in phosphorylation of MLC and FAK in human VSMCs.

In addition, we assessed Ca^2+^ influx in response to Ang II (Supplementary *Figure S9*). In the NT group, treatment with PP2 or melittin showed no significant changes in Ang II-induced Ca^2+^ influx. In contrast, in cells from HT only NOX5 inhibition significantly reduced Ca^2+^ transients induced by Ang II, suggesting a role for NOX5 in Ca^2+^ influx in cells from HT subjects.

### 3.8 NOX5/c-Src interplay is involved in VSMC cytoskeletal remodelling and migration in hypertension

**Figure 6:**
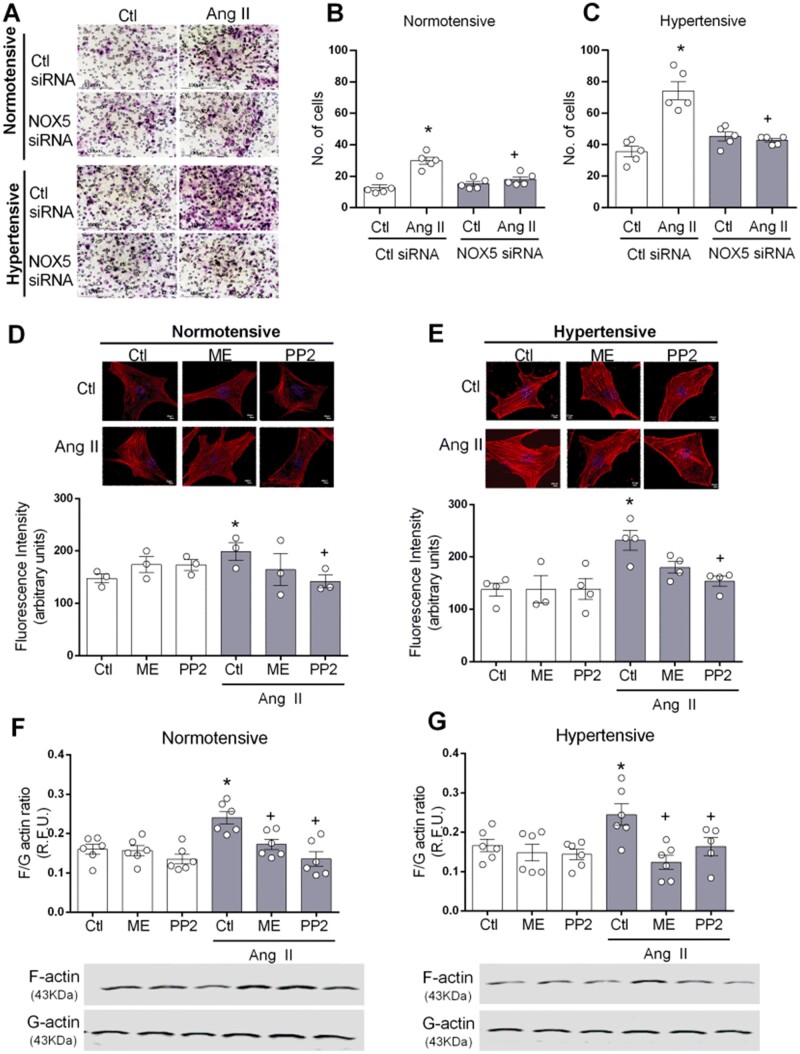
NOX5 and c-Src are involved in migration and actin polymerization in VSMC from HT subjects. Cells were stimulated with Ang II (100 nmol/L) in VSMCs transfected with NOX5 or control (Ctl) siRNA. Some experiments were performed in the presence and absence of melittin (NOX5 inhibitor, 10 nmol/L) and PP2 (Src inhibitor, 10 μmol/L). (*A*) Representative brightfield views of migrated cells from NT and HT groups. Objective: ×20. Scale bars: 150 μm. Quantification of migration in cells from NT (*B*) and HT (*C*) subjects. F‐actin‐specific phalloidin staining in VSMCs from NT (*D*) and HT (*E*) subjects. Objective: ×40. Scale bars: 20 μm. F/G-actin ratio was measured by western blot in cells from NT (*F*) and HT (*G*). Results are expressed as mean±SEM of 3–6 separate experiments. Statistical significance was determined by one-way ANOVA followed by Bonferroni’s post-test *P<0.05 vs. control (Ctl), ^+^*P*<0.05 vs. Ctl + Ang II.

c-Src is a versatile kinase involved in multiple cellular events underlying vascular remodelling, such as cytoskeletal remodelling and cell migration. Following our observation that in HT NOX5/c-Src affects several signalling pathways, we questioned the role of NOX5 and c-Src in actin polymerization and cellular migration in hypertension. Cells transfected with NOX5 siRNA were subjected to the Boyden chamber migration assay (*Figure 6A–C*). Ang II-induced migration in cells from both NT and HT subjects. NOX5 silencing reduced Ang II-induced migration in both groups.

Ang II induced an increase in actin polymerization assessed by F-actin staining in VSMC from NT and HT subjects, an effect blocked by treatment with PP2 (*Figure 6D and E*). Treatment with melittin reduced the staining in cells from HT. Similarly, the F/G-actin ratio was increased by Ang II in both groups (*Figure 6F and G*). NOX5 and Src inhibition decreased F/G-actin ratio in both groups. Together these results suggest that NOX5/c-Src regulates VSMC function by influencing cytoskeletal organization, which in the presence of oxidative stress in hypertension leads to cytoskeletal reorganization and VSMC migration.

### 3.9 c-Src is involved in vascular hypercontractility in transgenic NOX5 mice

**Figure 7:**
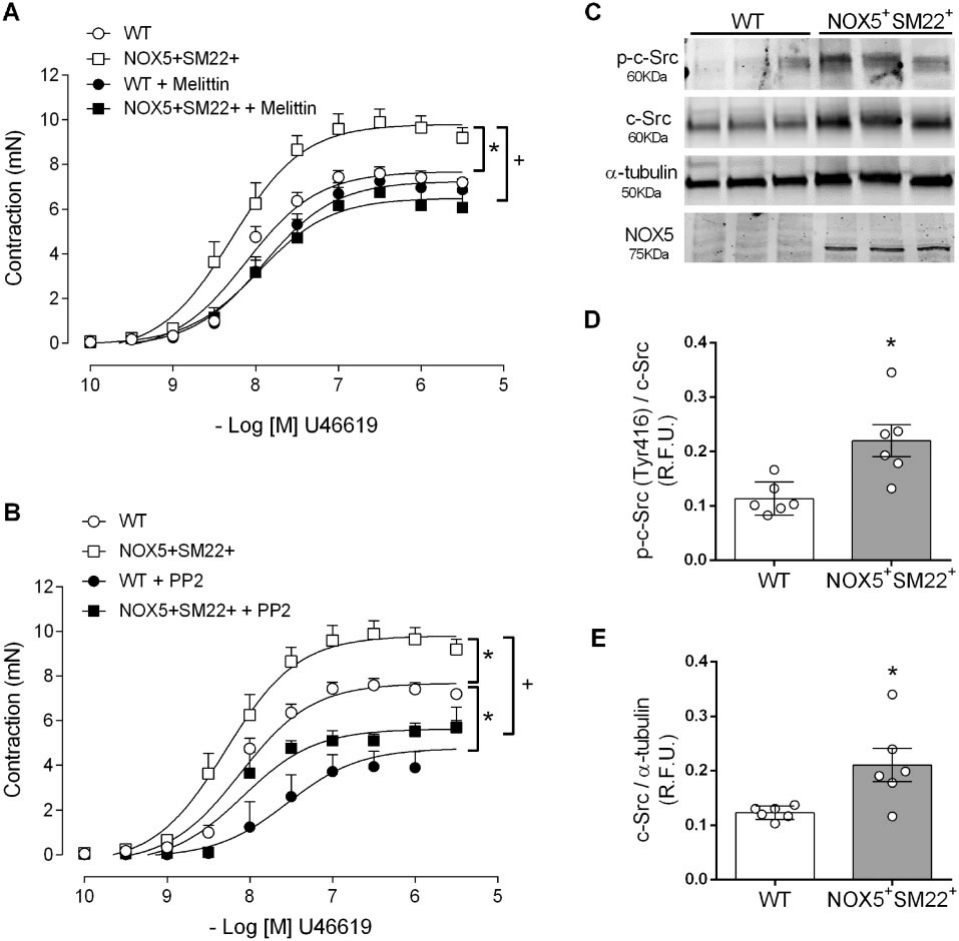
c-Src inhibition reduced contraction in NOX5 transgenic mice. Isolated mesenteric arteries from WT and VSMC-specific NOX5 transgenic mice (NOX5+SM22+) were used to assess vascular function by wire myography. Concentration–response curves to U46619 were performed in the presence or absence of melittin (*A*) and PP2 (*B*). U46619 responses were normalized by *E*_max_ of KCl 120 mmol/L. Expression of phosphorylated and total c-Src were assessed by western blot in mesenteric arteries from WT and NOX5 transgenic mice. NOX5 expression was assessed as control (*C*). Quantification of expression of phosphorylated (*D*) and total c-Src (*E*). Protein quantification was normalized by total c-Src or α-tubulin. Results are expressed as mean±SEM of 5–6 separate experiments. Statistical significance was determined by unpaired Student’s *t*-test for comparisons between two groups and two-way ANOVA followed by Bonferroni’s post-test for multiple comparisons. *P<0.05 vs. WT and ^+^*P*<0.05 vs. NOX5+SM22+.

As proof of concept, we investigated the functional significance of NOX5/c-Src signalling in mice expressing human NOX5 in a VSMC-specific manner and tested vascular reactivity in intact arteries. We previously showed that these mice have hyperreactive contractile responses to vasoconstrictors.^10^ Concentration–response curves to the thromboxane A2 analogue, U46619 were performed in mesenteric arteries isolated from WT and NOX5 transgenic mice (*Figure 7*). Vascular contraction induced by U46619 was increased in NOX5 mice compared with WT mice. Treatment with melittin significantly reduced contractile responses only in NOX5 mice, confirming the role of NOX5 in the hypercontractility observed in these mice (*Figure 7A*). c-Src inhibition with PP2 reduced contractile responses in WT mice (*Figure 7B*). In addition, Src inhibition was able to reduce the hypercontractile responses observed in NOX5 mice (*Figure 7B*), suggesting an important role for c-Src in NOX5-regulated vascular contraction. We also demonstrated that c-Src expression and phosphorylation are increased in mesenteric arteries from NOX5 mice (*Figure 7C–E*).

## 4. Discussion

A vascular hallmark of hypertension is structural remodelling and injury of resistance arteries due to de-differentiation and dysfunction of VSMCs, the major component of the vascular media.^24^^,^^25^ Molecular mechanisms underlying VSMC changes in human hypertension are elusive, but activation of stress-response pathways and dysregulated generation of NOX-derived ROS are important as we describe here. Specifically, we delineate NOX5 as a major source of O_2_^−^ and H_2_O_2_ in human VSMCs and demonstrate that in hypertension NOX5 is associated with activation of redox-sensitive signalling pathways, processes regulated by c-Src. Moreover, proof of concept studies in mice expressing human NOX5 in a VSMC-specific manner corroborates an important role for c-Src in NOX5-associated vascular hyperreactivity. We show that c-Src is activated in a NOX5-dependent manner and that NOX-induced ROS production is c-Src-dependent. Together our results define NOX5/ROS/c-Src as a novel signalling pathway in human VSMCs, a system that is augmented in hypertension contributing to VSMC cytoskeletal disorganization and vascular dysfunction.

NOX-derived ROS generation is now considered a major driver of vascular damage in hypertension.^6^^,^^24^^,^^25^ Extensive evidence in experimental models of hypertension indicates up-regulation of vascular NOX1, NOX2, and NOX4 as causes of oxidative stress and cardiovascular dysfunction and remodelling.^6^^,^^26–29^ Mice deficient in p22phox,^30^ p47phox,^31^ NOX1,^32^ and NOX2^28^^,^^33^ have blunted Ang II-induced hypertension and reduced hypertension-associated cardiovascular remodelling. Mice expressing NOX5 in a renal-specific manner are HT,^11^ and mice expressing NOX5 in VSMCs exhibit vascular dysfunction and hyperreactivity.^10^ Here, in human hypertension, we corroborate experimental evidence that VSMC NOX-derived ROS generation is increased, and clearly demonstrates that NOX5 up-regulation is a major underlying trigger. Expression of VSMC NOX4, but not NOX1 or NOX2, was also increased in hypertension. NOX4 is constitutively active and may contribute to increased basal ROS levels in VSMCs.^34^ On the other hand, under certain conditions, NOX4 has been suggested to be vasoprotective and the increase in VSMC expression, we observed in hypertension may represent a compensatory response to NOX5 up-regulation.^34^^,^^35^ Interactions between NOX4 and NOX5 have previously been suggested.^36^^,^^37^ Our findings highlighting NOX5 together with NOX4 in VSMC dysfunction in hypertension have clinical relevance because these NOX isoforms have been identified as novel BP-related genes.^13^ In addition, we assessed expression of p22phox, an essential subunit for activation of NOX1, NOX2, and NOX4. Several genetic polymorphisms of the p22phox gene have been reported to be associated with hypertension and other cardiovascular diseases that can impact NOX1–4 expression and activation.^38^ However, our results showed no differences in protein expression of p22phox in VSMC from NT and HT subjects, indicating that the up-regulation of the catalytic Nox subunits play an important role in VSMC alterations in hypertension.

Associated with NOX5-induced VSMC oxidative stress in hypertension was increased post-translational modification of downstream signalling targets. In particular peroxiredoxins (Prxs), important in cellular defence and peroxide signalling,^39^ and DJ-1, an oxidative stress-response protein,^40^ were hyperoxidized in HT VSMCs. Both Prxs and DJ-1 react directly with H_2_O_2_ and have critical cysteine residues highly susceptible to oxidation.^41^ High levels of H_2_O_2_ lead to formation of sulfinylated (-SO_2_H) and sulfonylated (-SO_3_H) forms of both Prxs and DJ-1,^40^^,^^42^ which we detected in VSMCs from HT patients. Of significance these proteins were irreversibly oxidized, which likely contributes to VSMC de-differentiation and end-stage cell damage. Associated with protein hyperoxidation in hypertension, we observed increased phosphorylation of PKC, ERK1/2, and MLC, important in VSMC contraction, growth, migration, and cytoskeletal organization.^3^^,^^25^ These processes appear to be NOX5- and redox-sensitive because NOX5 siRNA, melittin, SOD, and catalase attenuated responses in hypertension.

Molecular mechanisms linking NOX5 to downstream signalling elements in hypertension involve multiple systems, but c-Src may be especially important because (i) it acts as a signalling hub in VSMCs,^43^ (ii) it is tightly regulated by vasoactive and pro-HT factors, including Ang II,^44^^,^^45^ and (iii) c-Src polymorphisms have been associated with hypertension.^14^ Moreover c-Src is redox-sensitive and its activation by ROS involves sulfenylation of Cys-185 and Cys-277 to promote phosphorylation at Tyr416.^17^ Our results reveal that VSMC c-Src is up-regulated in hypertension through processes involving increased oxidation and phosphorylation. Functionally c-Src oxidation/phosphorylation is associated with activation of downstream signalling pathways because inhibition of c-Src blunted phosphorylation of MLC in Ang II-stimulated VSMCs from HT subjects. These findings underpin redox-regulated c-Src as a key element in VSMC signalling in human hypertension and support our previous studies in experimental models of hypertension and c-Src^+/^^−^ mice, where we demonstrated a role for c-Src and oxidative stress in Ang II-induced vascular dysfunction and hypertension.^15^

Cross-talk between c-Src and NOX has been demonstrated in various cell types.^46–48^ Here, we show that c-Src is tightly regulated by NOX-derived ROS in human VSMCs. In particular, NOX5 seems to be critically important because NOX5 siRNA and melittin prevented Ang II-stimulated phosphorylation of c-Src in both NT and HT VSMCs, whereas NOX1–4 inhibition, did not influence phosphorylation of c-Src. Supporting our findings others have demonstrated that NOX5 regulates c-Src through oxidation in human cancer cell lines.^50^ To our knowledge, the interplay we describe here between NOX5 and c-Src in human VSMC has not been previously demonstrated.

The central position of c-Src in vascular signalling is also evidenced by its role in being both a mediator and an effector of NOX-induced ROS production.^15^^,^^18^ In line with this, we found that ROS production in human VSMCs involves a Src-dependent process because PP2 inhibited Ang II-induced production of NADPH-dependent ROS and H_2_O_2_ in both NT and HT subjects. Mechanisms whereby c-Src influences NOX-ROS production may involve its role as a kinase to promote phosphorylation of target proteins. Supporting this, we previously showed that c-Src regulates NOX1/2 activity and oxidative stress in VSMCs by inducing phosphorylation of the NOX subunit p47phox.^18^ Similar to p47phox NOX5 contains several serine, threonine, and tyrosine residues that are prone to phosphorylation.^7^ PKC, Ca^2+^–calmodulin‐dependent protein kinase II and the tyrosine kinase c-Abl are known to phosphorylate NOX5.^49^^,^^50^ However, our results suggest that NOX5 is upstream of Ang II-induced c-Src activation. In this context, ROS generation in in human VSMCs may be orchestrated by the interplay between NOX isoforms and c-Src.

To explore the functional significance of the NOX5/ROS/c-Src axis, we examined migration and actin polymerization, processes involved in VSMC cytoskeletal organization and phenotypic differentiation. NOX5 silencing reduced migration in both NT and HT subjects. In addition, Ang II-induced actin polymerization was reduced by PP2 and melittin in both groups. These data suggest that regulation of human VSMC function involves c-Src and NOX5. In hypertension, when NOX5 activity and oxidative stress are increased, these processes are up-regulated influencing vascular dysfunction in hypertension.

To further interrogate the functional interplay between c-Src and VSMC NOX5 in intact vessels, we assessed vascular function in arteries from VSMC-specific NOX5 knockin mice. In line with the role of c-Src in vascular contraction,^16^^,^^51^ we observed a reduction in vascular reactivity in WT mice. We previously demonstrated that these NOX5 transgenic mice exhibit a hyperreactive vascular phenotype compared to controls.^10^ Here, we showed that agonist-induced hypercontractile responses in NOX5 mice were attenuated by PP2 and melittin, implicating a role for c-Src in NOX5-associated vascular dysfunction. Supporting this notion, phosphorylation and expression of c-Src were significantly increased in vessels from NOX5 transgenic mice, findings that were recapitulated in VSMCs from HT subjects that exhibited up-regulation of NOX5 and c-Src.

In conclusion, we identify NOX5 as a major source of oxidative stress and aberrant VSMC signalling in human hypertension. Molecular mechanisms linking NOX5 and downstream targets involve redox-sensitive c-Src, which is hyperactivated in hypertension. Our findings define NOX5/ROS/c-Src as a novel signalling pathway in human VSMCs and suggest a feedforward loop between NOX/ROS and redox-regulated c-Src, which is amplified in hypertension and which contributes to VSMC dysfunction. Dampening this signalling network may ameliorate VSMC injury and improve vascular dysfunction in hypertension.

## Supplementary material

Supplementary material is available at *Cardiovascular Research* online.

## Authors’ contributions

L.L.C. designed the study, performed experiments, analysed data, prepared the figures, and wrote the manuscript. A.C.M. performed experiments, analysed data, and provided critical discussion. Y.W., M.H., Z.Z., F.J.R., K.B.N., and R.A.-L. performed experiments. F.R.A. and T.G. contributed to critical discussion. The BCN-E-BCN probe for the sulfenylation assay was synthesized by T.J.G. in RCH’s laboratory. R.M.T. provided overall leadership and supervision, funding, designed the study, supported experiments, critical discussion, preparation, writing, and submission of the manuscript.

**Conflict of interest:** The authors declare that they have no conflit of interest.

## Funding

This work was funded by grants from the British Heart Foundation (BHF) (RG/13/7/30099, RE/18/6/34217) and the Medical Reseach Council (MRC) (MC-PC-15076). R.M.T. is supported through a BHF Chair award (CH/4/29762). A.C.M. is supported by a University of Glasgow Walton Fellowship. M.H. is supported by the Higher Education Commission (HEC), Pakistan.

## Data availability

The data that support the findings of this study are available from the corresponding author upon reasonable request. Please see Supplementary material online for detailed methods.

## Supplementary Material

cvab171_Supplementary_Data

## Notes

Translational Perspective Oxidative stress is a major factor contributing to vascular damage in hypertension. We corroborate experimental evidence that NOX-derived ROS generation is increased in human VSMCs and demonstrate that in human hypertension NOX5 up-regulation is a major trigger of VSMC dysfunction. We uncover new regulatory molecular mechanisms of NOX5 and define NOX5/ROS/c-Src as a novel signalling pathway in human VSMCs. This system is augmented in hypertension contributing to abnormal VSMC redox signalling, cytoskeletal disorganization and vascular dysfunction. Modulating the NOX5/ROS/c-Src pathway may have therapeutic potential by targeting redox signalling pathways involved in vascular dysfunction associated with hypertension.
