# Supplementary material for: Central role of c-Src in NOX5- mediated redox signalling in vascular smooth muscle cells in human hypertension
Source: Cardiovasc Res. 2021 Jul 28;118(5):1359–73. doi: 10.1093/cvr/cvab171 (PMC8953456; doi:10.1093/cvr/cvab171)
Supplement: cvab171_Supplementary_Data [file cvab171_supplementary_data.docx]

**SUPPLEMENTARY MATERIAL**

**Central role of c-Src in NOX5- mediated redox signaling in vascular smooth muscle cells in human hypertension**

Livia L Camargo PhD^1^, Augusto C. Montezano PhD^1^, Misbah Hussain PhD^2^, Yu Wang MSc^1^, Zhiguo Zou MSc^1^, Francisco J Rios PhD^1^, Karla B Neves PhD^1^, Rheure Alves-Lopes PhD^1^, Fazli R Awan PhD^2^, Tomasz J Guzik MD, PhD^1^, Thomas Jensen MSci^3^,

Richard C. Hartley PhD^3^, Rhian M. Touyz MD, PhD^1^

^1^ Institute of Cardiovascular and Medical Sciences, University of Glasgow; ^2^ Diabetes and Cardio-Metabolic Disorders Laboratory, Health Biotechnology Division, National Institute for Biotechnology and Genetic Engineering (NIBGE), Faisalabad, Pakistan; ^3^WestCHEM School of Chemistry, University of Glasgow.

**Correspondence:**

Rhian M Touyz MBBCh, PhD and Livia L Camargo PhD

Institute of Cardiovascular & Medical Sciences

University of Glasgow

126 University Place, Glasgow G12 8TA

Tel: + 44 (0)141 330 7775/7774

Email: [Rhian.Touyz@glasgow.ac.uk](mailto:Rhian.Touyz@glasgow.ac.uk); Livia.DeLuccaCamargo@glasgow.ac.uk

**Supplementary Methods**

Ethics approval was obtained from the West of Scotland Research Ethics Service (WS/12/0294) and the research ethics board of the Ottawa Hospital Research Institute (OHRI), Canada (#997392132). Written informed consent was obtained for all study participants in accordance with the Declaration of Helsinki. All experimental protocols on mice were performed in accordance with the Ethical Principles in Animal Experimentation adopted by the West of Scotland Research Ethics Service and in accordance with the United Kingdom Animals Scientific Procedures Act 1986 and ARRIVE Guidelines and approved by the institutional ethics review committee (70/9021).

**Human vascular tissue**

Vascular tissue was obtained from normotensive (NT) (n=13) and hypertensive subjects (HT) (n=13) undergoing elective maxillofacial surgery at the Craniofacial/Oral and Maxillofacial Unit, Queen Elizabeth University Hospital, Glasgow (Supplementary Table 1). Following surgery, isolated small arteries (150-300 µM in diameter) dissected from excess surgical tissue from the neck or face were cleaned from adipose tissue and processed for western blot as we previously described^1^. Hypertension was defined as systolic blood pressure >140/90 mmHg or a history of hypertension on antihypertensive treatment, according to clinical notes. Whole vascular tissue was used to assess expression of NOX isoforms by western blotting.

Gluteal biopsies of subcutaneous fat measuring approximately 2 cm × 1 cm × 1 cm were obtained under local anaesthetic from NT (n=10) and HT (n=5) volunteers at the Ottawa Hospital Research Institute (Supplementary Table 2). Small arteries were dissected from these biopsies and used for primary culture of VSMCs. Hypertension was defined as systolic blood pressure >140/90 mmHg or a history of hypertension on antihypertensive treatment.

**Primary culture of vascular smooth muscle cells from human small arteries**

Primary VSMCs were isolated from small arteries obtained from gluteal biopsies, by enzymatic digestion, as we previously described^1^. Briefly, excess fat, connective tissue and adventitia from isolated arteries were stripped off, and vessels were placed in Ham’s F-12 culture medium containing 1% gentamicin, collagenase (type 1), elastase, soybean trypsin inhibitor, and BSA, and were incubated for 1 hour at 37°C under constant agitation. The digested tissue was further dissociated by repeated aspiration through a syringe with a 20-gauge needle. The cell suspension was centrifuged (2000 rpm, 4 minutes) and the cell pellet was resuspended in Ham’s F-12 culture medium containing 10% FBS. Cells were seeded onto 25-mm round glass coverslips coated with Matrigel basement membrane matrix (Becton Dickinson Labware). For the first 48 hours, cells were incubated in Ham’s F-12 culture medium containing 10% heat-inactivated foetal calf serum. Thereafter, VSMCs were maintained in DMEM supplemented with Smooth Muscle Growth Supplement (SMGS; Thermo Fisher Scientific) and 5% antibiotics (100 IU/ml penicillin – 100 μg/ml streptomycin)). Cell phenotype was confirmed by western blot of alpha smooth muscle actin (α -SMA) and fibroblast activator protein alpha (FAP- α) as markers of VSMC and fibroblasts, respectively. Human fibroblasts were used as positive control for FAP- α antibody (Supplementary Figure 1). At 80-90% confluence cells were treated with 0.025% trypsin-EDTA and passaged. VSMCs were studied at passage 4-7. Before experimentation, cells were rendered quiescent by overnight serum deprivation. For some experiments cells were pre-treated (30 mins) with melittin (NOX5 inhibitor, 100 nmol/L; Sigma-Aldrich) or PP2 (Src inhibitor, 10 μmol/L; Sigma-Aldrich). Then, cells were stimulated with Ang II for 5 min (100 nmol/L; Sigma-Aldrich).

**VSMC‐specific NOX5 transgenic mice**

We used VSMC-specific NOX5-expressing mice to assess whether c-Src inhibition influences vascular function. The transgenic NOX5 mice have been previously characterized and described^2^. Briefly, NOX5 mice (NOX5^+^SM22^+^, on an FVB background) express human NOX5β selectively in VSMCs. Mice were housed in individual cages in a room with controlled humidity and temperature (22°C–24°C), and in light/dark cycles of 12 hours with free access to food and tap water. Animals were euthanized by overdose of anaesthetic gas (isoflurane 3% plus 1 L/min O_2_) followed by cervical dislocation. They were studied at 20 weeks of age and tissue was collected for experiments. Small mesenteric arteries were used to assess vascular function by wire myography. Mesenteric arteries were used to investigate NOX5 dependent signalling.

**NOX5 and 22phox downregulation with siRNA**VSMCs from NT and HT subjects (8 x 10^5^ cells/well) were plated and cultured for 24h in growth medium (DMEM supplemented with SMGS and 5% antibiotics). VSMCs were incubated with 50 nmol/L of NOX5 siRNA (Stealth RNAi™ siRNA, Thermo Fischer Scientific) or 20 nmol/L of p22phox siRNA (Stealth RNAi™ siRNA, Thermo Fischer Scientific) complexed with Lipofectamine™ RNAiMAX (Thermo Fischer Scientific) as transfection reagent in DMEM without serum and antibiotics for 6 h. A sequence not homologous to any gene in the vertebrate transcriptome was used as control siRNA (Stealth RNAi™ siRNA Negative Control Lo GC, Thermo Fischer Scientific). After transfection, medium was replaced by growth medium (DMEM with SMGS and 5% antibiotics) and experiments were conducted 12-48 h after transfection.

**Measurement of NADPH-dependent O_2_^-^ generation**

Lucigenin-enhanced chemiluminescence assay was used to detect NADPH-dependent ROS generation in VSMC as we previously described^3^. Briefly cells were homogenized in lysis buffer and incubated with lucigenin (5 µmol/L, Sigma-Aldrich) and NADPH (0.1 mmol/L; Sigma-Aldrich). Luminescence was measured for 29 cycles of 1.8 seconds each by a luminometer (Lumistar Galaxy; BMG Labtech). Basal readings were recorded prior to the addition of NADPH as the substrate and were subtracted from the NADPH-dependent luminescence signal. ROS production was expressed as relative luminescence unit (RLU)/µg protein.

**Measurement of vascular H_2_O_2_**

Hydrogen peroxide (H_2_O_2_) levels in VSMC were assessed using the Amplex Red Hydrogen Peroxide/Peroxidase Assay Kit (Molecular Probes, Life Technologies) according to manufacturer´s instructions. H_2_O_2_ levels were calculated based on standard H_2_O_2_ curves and normalised to the protein concentration for each sample. The results are expressed in arbitrary units/milligram protein.

**Measurement of O_2_•^-^ by electron paramagnetic resonance**

Superoxide anion (O_2_•^-^) was measured by electron paramagnetic resonance^4^. Production of O_2_•^-^ was measured in in Krebs–Hepes buffer (99 mM NaCl, 4.69 mM KCl, 2.5 mM CaCl_2_.2H_2_O, 1.2 mM MgSO_4_.7H_2_O, 25 mM NaHCO_3_, 1.03 mM KH_2_PO_4_, 5.6 mM D (+) glucose, 20 mM HEPES, pH 7.4) containing chelating agents, deferoxamine (25 µM, Sigma) and Na diethyldithiocarbamate trihydrate (5 µM, Sigma). Confluent cells were incubated with hydroxylamine spin probe 1-hydroxy-3-methoxycarbonyl-2,2,5,5-tetramethylpyrrolidine (CM-H, 500 µM) for 10 min at 37°C, and then washed with PBS. Then cells were collected and placed into glass capillary tubes (Hirschmann Laborgeräte, Eberstadt, Germany) and subsequently assessed in an EPR spectrometer (e-scan R Bruker BioSpin GmbH). Oxidation of CMH by O_2_•^-^ results in formation of the stable nitroxide radical, 3-methoxy-carbonyl (CM). Therefore, the amount of CM formed equals the concentration of the reacting oxidant species. The concentration of CM was determined from the amplitude of the low field component of EPR spectra according to a calibration curve, generated using standard solutions of the 3-carboxy-proxyl (CP) radical (Noxygen). CP standard solutions of 1 µM, 5 µM and 10 µM were prepared from a 1 mM stock solution of CP dissolved in Krebs-HEPES buffer. Counts were recorded once a minute for 10 minutes and O_2_•^-^ formation recorded as µmol/minute. EPR spectra and kinetics were recorded from cell suspensions in 50 µl. Instrument settings were: centre field, 3375 G; modulation amplitude, 2.27 G; sweep time, 5.24 seconds; sweep width, 60 G; and 10 scans.

**Immunoblotting**

Total protein from vascular tissue and cells was extracted in lysis buffer containing Tris (50 mmol/L, pH 8.0), NaCl (150 mmol/L), Triton X-100 (1%), SDS (0.1%), supplemented with phenylmethylsulfonyl fluoride (PMSF; 1 mmol/L), pepstatin A (1 µg/mL), leupeptin (1 µg/mL), aprotinin (1 µg/mL), sodium fluorate (10 mmol/L) and sodium orthovanadate (1 mmol/L). Total protein lysate was sonicated, cleared by centrifugation at 12,000 rpm, at 4°C for 5 min and the pellet was discarded. Supernatants were collected and protein concentration was determined using the BCA Protein Assay kit (Thermo Fischer Scientific). Equal amounts of protein were resolved by SDS-PAGE and transferred onto a nitrocellulose membrane. Nonspecific binding sites were blocked with either non-fat dry milk or BSA, before overnight incubation in protein-specific primary antibodies. Fluorescence-coupled antibodies (LICOR) were incubated for 1 h and were visualized by an infrared laser scanner (Odyssey Clx, LICOR).

Western blotting images were quantified using the software Image Studio™ Lite. Protein expression levels were normalized to loading controls and expressed as absolute values or percentage (%) of the control. Primary antibodies were as follows: primary antibody used for NOX5 detection was kindly provided by Prof. David G. Harrison (Vanderbilt University); antibodies towards phosphorylated c-Src (p-c-Src) at Tyr416 and Tyr526, protein kinase C (p-PKC, Thr638/641), extracellular signal-regulated kinase 1/2 (p-ERK 1/2, Thr202/Tyr204), myosin light chain (p-MLC, Ser19) and focal adhesion kinase (p-FAK; Tyr397) were from Cell Signalling Technologies; total PKC, NOX2, NOX4, p22phox, phosphorylated serine/threonine/tyrosine (pSer/Thr/Tyr) and α-tubulin were from Abcam; NOX1 and β-actin antibodies were from Sigma-Aldrich and total c-Src antibody was from Santa Cruz Biotechnology.

**Assessment of protein sulfenylation**

To assess one of the first reversible cysteine oxidative modifications in proteins, sulfenylation, we used BCN-E-BCN, a cell permeable probe composed of two symmetrical strained cyclooctynes connected by a short ethylenediamine-derived linker that specifically binds the sulfenic acid groups in proteins^5^. Cells were scraped in lysis buffer supplemented with BCN-E-BCN (1 mmol/L), N-methylmalemide (10 mmol/L), catalase (200 U) and protease inhibitors (1 mmol/L PMSF and 1 µg/mL of aprotinin, leupeptin and pepstatin). Samples were kept on ice for 30 min and centrifuged at 16,000 x g for 4 min at 4˚C. BCN-E-BCN was conjugated with biotin using a copper-free click reaction. For this, lysates were incubated with azide-PEG3-biotin (1 mmol/L; Sigma-Aldrich). Excess azide-PEG3-biotin was removed by precipitating lysates in 100% ice cold acetone. Protein pellets were re-suspended in lysis buffer and prepared for western blot and protein levels were determined using the BCA Protein Assay kit (Thermo Fischer Scientific). Equal amounts of protein (500 µg) were added to a 50 µl slurry of nonliganded support beads (sepharose CL-4B beads, Sigma-Aldrich) to remove proteins with a tendency to bind non-specifically and incubated for 2 h at 4˚C with constant rotation. Beads were centrifuged at 1,000 x g for 2 min, the supernatant was collected and incubated with streptavidin beads (High-Capacity Streptavidin–Agarose Resin, Thermo Scientific) overnight at 4˚C with constant rotation. After the incubation steps beads were centrifuged at 1,000 x g for 2 min and washed with PBS three times. Proteins were then eluted in 50 µl of 2 x sample buffer for western blotting and boiled (5 min).

**Determination of irreversible DJ-1 and Prx oxidation**

VSMCs were lysed and analysed by immunoblotting using specific antibodies, which recognize the hyperoxidized catalytic centre (-SO_3_H) on DJ-1 (DJ-1 oxidized, Abcam) and Prx (Prx-SO3, Abcam).

**Calcium (Ca^2+^) influx**

Intracellular Ca^2+^ levels were measured in VSMCs from NT and HT subjects using the fluorescent Ca^2+^ indicator, Cal-520 acetoxymethyl ester (Cal-520/AM; Abcam; 10 μmol/L). Fluorescence measurements were performed using an inverted epifluorescence microscope (Axio Observer Z1 Live-Cell imaging system, Zeiss) with excitatory wavelengths of 490 and 488 nm. Images were acquired and analysed using Zen Pro Program (Zeiss). Cells were grown in 6-well plates and following the removal of culture media were incubated with 10 μmol/L of Cal-520 AM in 0.5% FBS at 37°C for 75 minutes followed by 30 minutes at room temperature. Following incubation, the dye solution was replaced with HEPES physiological saline solution contained the following components (in mM): NaCl 130, KCl 5, CaCl 1, MgCl 1, HEPES 20, and D-glucose 10, pH 7.4) for 30 minutes prior to imaging. Fluorescence intensity as a measure of [Ca^2+^]i, was monitored for 1 minute in basal condition and 2 minutes under Ang II (100 nmol/L) stimulation. In some experiments VSMCs were pre-treated for 30 minutes with melittin (100 nmol/L), PP2 (10 µmol/L), superoxide dismutase polyethylene glycol (PEG-SOD, 100 U/ml, Sigma-Aldrich) and catalase polyethylene glycol **(**PEG-catalase, 1000 U/ml; Sigma-Aldrich).

**Cell Migration**

VSMCs migration was assessed by a modified Boyden chamber assay using transwell 24-well cell culture inserts (8 μm pores size gelatinized polycarbonate membrane, Startedt). VSMCs were harvested and added to the insert (5 × 10^4^ cells/well), and culture medium with or without Ang II (100 nmol/L) was added to the well containing insert. After 16-hour incubation at 37°C, the top of the membrane was scraped with a cotton swab, leaving the cells that migrated through the membrane to the lower side. Then, cells adherent to the polycarbonate membrane were fixed with 95% ethanol and stained with Giemsa. The number of migrated cells on the lower face of the filter was counted in 10 random microscopic fields under a 20x magnification. (Observer Z1, Zeiss) using Image J software (National Institutes of Health, USA).

**Phalloidin staining**

Cytoskeletal organization was assessed using phalloidin staining (Cytoskeleton) according to the manufacturer’s instructions. Briefly, VSMCs were grown in 4-chamber slides coated with gelatine. At 50-60% confluency cells were rendered quiescent overnight using DMEM with 0.5% FBS. Cells were then incubated with Ang II (100 nmol/L. 60 min) in the presence and absence of inhibitors including melittin (100 nmol/L) and PP2 (10 µmol/L). After incubation, cells were fixed with 4% paraformaldehyde (PFA) in PBS at room temperature for 20 minutes. Fixed cells were washed 3 times with PBS, and permeabilized with 0.1% TritonX-100 for 10 minutes. Nonspecific binding sites were blocked with 1% BSA in PBS for 30 minutes at room temperature followed by incubation with phalloidin stain (100 nmol/L) in PBS with 1% BSA for 30 minutes in a humidified chamber in the dark. Cells were then washed 3 times with PBS and mounted using Prolong medium with DAPI (Thermo Fisher Scientific). Fluorescence imaging was performed using a 40x plan dry lens in a LSM500 confocal imaging system (Zeiss). DAPI was excited at 405 nm and phalloidin at 535 nm. Images were acquired using Zen Pro (Zeiss). Each experimental group was imaged with a minimum of 10 images for 3 different experiments. For quantification of F-actin intensity, region of interest (ROI) was defined around each cell, and average F-actin intensity per pixel was then analysed by ImageJ software (National Institutes of Health, USA).

**G-actin/F-actin Assay**

Filamentous actin (F-actin) and free globular-actin (G-actin) content were assessed from lysates of cells from NT and HT subjects according to the manufacturer’s recommendations (G-actin/F-actin in vivo Assay, Cytoskeleton). Briefly, cells were lysed in F-actin stabilization supplemented with ATP (1 mM) and a protease inhibitor cocktail (pepstatin, leupeptin, benzamidine, TAME) and incubated at 37⁰C for 10 minutes. Lysed cells were then centrifuged at 100,000 rpm at 37⁰C for 60 minutes. Cytoplasmic fractions (supernatant) were separated for analysis of G-actin. F-actin was obtained from the cell pellet resuspended in F-actin depolymerization buffer and incubated 1 hour on ice. G-actin and F-actin samples were analysed by western blot using anti-actin antibody (provided in the kit). The ratio of G-actin to F-actin was quantified by scanning densitometry were quantified using the software Image Studio™ Lite.

**Vascular function assessed by wire myography**

Mesenteric resistance arteries from wild type (WT) and NOX5 transgenic mice (NOX5^+^SM22^+^) were dissected as previously described^6^. Briefly, arterial segments were mounted on isometric wire myographs (Danish Myo Technology) filled with 5 mL of physiological saline solution (in mmol/L: 130 NaCl, 14.9 NaHCO_3_, 4.7 KCl, 1.18 KH_2_PO_4_, 1.17 MgSO_4_•7H_2_O, 5.5 glucose, 1.56 CaCl_2_•2H_2_O, and 0.026 EDTA) and continuously gassed with a mixture of 95% O_2_ and 5% CO_2_ while being maintained at a constant temperature of 37°C ± 0.5°C. Following 60 minutes of equilibration, the contractile responses of arterial segments were assessed by the addition of KCl (62.5 mmol/L) to the organ baths. U46619 (thromboxane A2 analogue) concentration-response curves were generated to evaluate vasoconstriction in arteries form WT and NOX5 mice. In some experiments, arteries were preincubated for 30 minutes with melittin (NOX5 inhibitor, 100 nmol/L; Sigma-Aldrich) or PP2 (Src inhibitor, 10 μmol/L; Sigma-Aldrich).

**Reagents**

Lucigenin, angiotensin II, N-ethylmaleimide (NEM), sodium orthovanadate (Na_3_VO_4_), catalase, elastase, soybean trypsin inhibitor, PMSF, aprotinin, leupeptin, pepstatin, azide-PEG3-biotin conjugate, superoxide dismutase polyethylene glycol, and catalase polyethylene glycol were from Sigma-Aldrich. Collagenase type-1 was from Worthington Biochemical Corporation. Sodium fluorate was from AnalaR Normapur.

**Statistical Analysis**

All results are reported as mean±SEM. For comparisons between two groups t-test was used. For multiple comparisons one-way or two-way analysis of variance (ANOVA) followed by Bonferroni’s post-test was conducted where appropriated. Graphs were plotted in GraphPad Prism 8 software. *p*<0.05 were considered significant.

**References**

1. Montezano AC, Lopes RA, Neves KB, Rios F and Touyz RM. Isolation and Culture of Vascular Smooth Muscle Cells from Small and Large Vessels. *Methods Mol Biol*. 2017;**1527**:349-354.

2. Montezano AC, De Lucca Camargo L, Persson P, Rios FJ, Harvey AP, Anagnostopoulou A, Palacios R, Gandara ACP, Alves-Lopes R, Neves KB, Dulak-Lis M, Holterman CE, de Oliveira PL, Graham D, Kennedy C and Touyz RM. NADPH Oxidase 5 Is a Pro-Contractile Nox Isoform and a Point of Cross-Talk for Calcium and Redox Signaling-Implications in Vascular Function. *Journal of the American Heart Association*. 2018;**7**.

3. Camargo LL, Harvey AP, Rios FJ, Tsiropoulou S, Da Silva RNO, Cao Z, Graham D, McMaster C, Burchmore RJ, Hartley RC, Bulleid N, Montezano AC and Touyz RM. Vascular Nox (NADPH Oxidase) Compartmentalization, Protein Hyperoxidation, and Endoplasmic Reticulum Stress Response in Hypertension. *Hypertension*. 2018;**72**:235-246.

4. Dikalov SI, Kirilyuk IA, Voinov M and Grigor'ev IA. EPR detection of cellular and mitochondrial superoxide using cyclic hydroxylamines. *Free radical research*. 2011;**45**:417-430.

5. McGarry DJ, Shchepinova MM, Lilla S, Hartley RC and Olson MF. A Cell-Permeable Biscyclooctyne As a Novel Probe for the Identification of Protein Sulfenic Acids. *ACS Chem Biol*. 2016;**11**:3300-3304.

6. Schiffrin EL, Park JB, Intengan HD and Touyz RM. Correction of arterial structure and endothelial dysfunction in human essential hypertension by the angiotensin receptor antagonist losartan. *Circulation*. 2000;**101**:1653-1659.

**Supplementary Table 1. Characteristics of subjects from whom intact small arteries were obtained.**

| **Parameters** | **NT** | **HT** | **P value** |
| --- | --- | --- | --- |
| N | 13 | 13 |  |
| Sex (F/M) | 6/7 | 7/6 |  |
| Age (years) | 63.5 ± 2.78 | 69.3 ± 2.73 | 0.1507 |
| SBP (mmHg) | 128.8 ± 3.15 | 143.8 ± 5.8 | 0.0305* |
| DBP (mmHg) | 72.8 ± 2.47 | 81.08 ± 1.97 | 0.0089* |

**Supplementary Table 2. Characteristics of subjects from whom small arteries were isolated for VSMC primary culture.**

| **Parameters** | **NT** | **HT** | **P value** |
| --- | --- | --- | --- |
| N | 10 | 5 |  |
| Sex (F/M) | 7/3 | 2/3 |  |
| SBP (mmHg) | 119.1 ± 2.98 | 146.2 ± 6.03 | 0.0006* |
| DBP (mmHg) | 73.3 ± 2.61 | 92.6 ± 3.07 | 0.0006* |
| Heart rate (bpm) | 66 ± 2.09 | 69 ± 4.06 | 0.5041 |
| Sodium (mmol/L) | 138.8 ± 0.51 | 139.1 ± 0.63 | 0.6778 |
| Chloride (mmol/L) | 102.5 ± 0.79 | 103.9 ± 1.29 | 0.3596 |
| Potassium (mmol/L) | 4 ± 0.1 | 3.8 ± 0.15 | 0.1550 |
| Renin (ng/L) | 7 ± 1.62 | 9.92 ± 3.94 | 0.4639 |
| Aldosterone (pmol/L) | 194.3 ± 21.44 | 352.3 ± 91.07 | 0.0627 |
| Aldosterone/Renin ratio | 35.3 ± 6.08 | 51.85 ± 18.57 | 0.3466 |
| Cholesterol (mmol/L) | 4.6 ± 0.14 | 4.3 ± 0.28 | 0.3971 |
| Triglycerides (mmol/L) | 1.2 ± 0.18 | 0.96 ± 0.06 | 0.2270 |
| HDL (mmol/L) | 1.43 ± 0.06 | 1.44 ± 0.09 | 0.9160 |
| LDL (mmol/L) | 2.62 ± 0.18 | 2.5 ± 0.27 | 0.7105 |
| TC:HDL | 3.2 ± 0.19 | 3 ± 0.23 | 0.5179 |
| BMI (kg/m^2^) | 27.3 ± 1.12 | 31 ± 2.08 | 0.1060 |
| Height (cm) | 170.5 ± 2.3 | 168.3 ± 3.38 | 0.5984 |
| Weight (cm) | 79.5 ± 3.96 | 80.5 ± 11.1 | 0.9266 |
| Waist circunference (cm) | 86.14 ± 5.0 | 102.7 ± 5.27 | 0.0444* |
| Hip circunference (cm) | 108.9 ± 3.42 | 111.9 ± 4.73 | 0.6038 |

**Supplementary Figure 1. Expression of VSMC and fibroblast markers in primary cell culture from normotensive (NT) and hypertensive (HT) subjects.** Fibroblast activator protein alpha (FAPα) and alpha smooth muscle actin (α-SMA) were detected by western blot in VSMC cells from NT and HT subjects. Human fibroblasts (Fibro) were used as a positive control for FAPα. α-tubulin was used as a loading control.

**

**

**Supplementary Figure 2. ROS generation in the presence of antioxidants.** Cells were stimulated with angiotensin II (Ang II, 100 nmol/L) for 5 minutes in the presence and absence of pegylated superoxide dismutase (PEG-SOD; 100U/ml) or pegylated catalase (PEG-CAT, 100U/ml). Superoxide anion was measured by EPR in cells from NT (A) and HT (B) subjects. NADPH dependent ROS was measured by lucigenin-derived chemiluminescence in cells from NT (A) and HT (B) subjects. Hydrogen peroxide was assessed by amplex red in VSMC from NT (E) and HT (F). Results are expressed as mean ±SEM of n=2-7. Statistical significance was determined by Student’s t-test for comparisons between two groups and one-way ANOVA followed by Bonferroni’s post-test for multiple comparisons. *p<0.05 vs CTL; ^+^p<0.05 vs CTL+Ang II.

**

**

**Supplementary Figure 3. Expression of p22phox, the NADPH oxidase subunit that associates with NOX1-4, is not changed in VSMC from HT subjects.** Expression of p22phox was assessed by western blot in cells from NT and HT subjects. α-tubulin was used as loading control. Results are expressed as mean ±SEM of n=6.





**Supplementary Figure 4. Redox signaling and contractile machinery are upregulated in VSMC from hypertensive subjects.** Phosphorylation of c-Src at Tyr 416 (A) and Tyr 527 (B), PKC (C), MLC (D) and ERK1/2 (E) were detected by western blot in cells from NT and HT subjects. Total c-Src, PKC or α-tubulin were used as loading control. Representative tracings (F) and are under the curve (G) of Ang II induced Ca^2+^ influx measured in VSMCs from NT and HT subjects. Cal-520 fluorescence was measured as the change in fluorescence relative to baseline fluorescence (∆F/F_0_). Results are expressed as mean±SEM of 6-9 separate experiments. Statistical significance was determined by unpaired Student’s t-test. *p<0.05 vs NT.





**Supplementary Figure 5. Role of ROS in Ang II-mediated signaling in human VSMC.** Cells were stimulated with angiotensin II (Ang II, 100 nmol/L) for 5 minutes in the presence and absence of pegylated superoxide dismutase (PEG-SOD; 100U/ml) and pegylated catalase (PEG-CAT, 100U/ml). Phosphorylation of c-Src at Tyr 416 (A and B, respectively), PKC (C and D, respectively) and MLC (E and F, respectively) were detected by western blot in cells from NT and HT subjects. Total c-Src, PKC or α-tubulin were used as loading control. Statistical significance was determined by one-way ANOVA followed by Bonferroni’s post-test. Results are expressed as mean±SEM of 4-7 separate experiments. *p<0.05 vs CTL; ^+^p<0.05 vs CTL+Ang II.





**Supplementary Figure 6.** **Ang II induced Ca^2+^ influx is ROS dependent in VSMC from HT subjects.** Ca^2+^ influx was measured by Cal520-AM fluorescence in the presence of Ang II (100 nmol/L). Representative tracings of Ca^2+^ influx in the presence of PEG-SOD in cells from NT (A) and HT (B) subjects. Representative tracings of Ca^2+^ influx in the presence of PEG-Catalase in cells from NT (C) and HT (D) subjects. Bar graph represents area under the curve (AUC) for NT (E) and HT (F) groups. Cal-520 fluorescence was measured as the change in fluorescence relative to baseline fluorescence (∆F/F_0_). Results are expressed as mean±SEM of 5-6 separate experiments. Statistical significance was determined by unpaired Student’s t-test. *p<0.05 vs control (Ctl).

**

**

**Supplementary Figure 7. c-Src is involved in ROS generation in human VSMCs.** Cells were stimulated with angiotensin II (Ang II, 100 nmol/L) for 5 minutes in the presence and absence of PP2 (10 μmol/L, Src inhibitor). NADPH dependent ROS and hydrogen peroxide were measured by lucigenin-derived chemiluminescence and amplex red in VSMCs from NT (A and B, respectively) and HT (C and D, respectively) subjects. Results are expressed as mean±SEM of 7-8 separate experiments. Statistical significance was determined by one-way ANOVA followed by Bonferroni’s post-test. *p<0.05 vs control (Ctl) and ^+^p<0.05 vs Ctl + Ang II.





**Supplementary Figure 8**. **Role of NOX1-4 in Ang II-induced MLC activation in human VSMC.** p22phox silenced cells were stimulated with Ang II (100 nmol/L) for 5 minutes in the presence and absence of melittin (ME) (10 nmol/L) and PP2 (10 μmol/L). Phosphorylated MLC expression detected by western blot in cells from NT (A) and HT (B) subjects. α-tubulin was used as loading control. Results are expressed as mean±SEM of 5-9 separate experiments. Statistical significance was determined by two-way ANOVA. *p<0.05 vs control (Ctl), ^#^p<0.05 vs p22; ^+^p<0.05 vs Ctl + Ang II and ^&^p<0.05 vs p22 + Ang II.





**Supplementary Figure 9. Effect of NOX5 and c-Src inhibition on Ang II induced Ca^2+^ influx in human VSMC.** Ca^2+^ influx was measured by Cal520-AM fluorescence in the presence of Ang II (100 nmol/L). Representative tracings of Ca^2+^ influx in the presence of melittin (10 nmol/L) in cells from NT (A) and HT (B) subjects. Representative tracings of Ca^2+^ influx in the presence of PP2 (10 μmol/L) in cells from NT (C) and HT (D) subjects. Bar graph represents area under the curve (AUC) for NT (E) and HT (F) groups. Cal-520 fluorescence was measured as the change in fluorescence relative to baseline fluorescence (∆F/F_0_). Results are expressed as mean±SEM of 5-6 separate experiments. Statistical significance was determined by unpaired Student’s t-test. *p<0.05 vs control (Ctl).
